# Editorial: Novel approaches to predict and improve sperm function during semen storage

**DOI:** 10.3389/fvets.2024.1527644

**Published:** 2024-12-20

**Authors:** José Luis Ros-Santaella, Francisco Alberto García-Vázquez, Federica Turri, Eliana Pintus

**Affiliations:** ^1^Department of Veterinary Sciences, Faculty of Agrobiology, Food and Natural Resources, Czech University of Life Sciences Prague, Prague, Czechia; ^2^Department of Physiology, Faculty of Veterinary Science, University of Murcia, Murcia, Spain; ^3^Institute of Agricultural Biology and Biotechnology (IBBA), National Research Council (CNR), Lodi, Italy

**Keywords:** antioxidants, artificial insemination, biomarkers, domestic animals, oxidative stress, semen analyses, semen preservation, ultrasonography

Artificial insemination (AI) is the most popular assisted reproductive technology (ART) applied to domestic animals thanks to the advantages that it provides compared to natural mating (1). In addition, ARTs represent a powerful tool in conservation breeding programs to save wild endangered species from extinction (2). Because sperm quality is critical for ARTs success, different approaches like semen refrigeration (liquid or encapsulated) and cryopreservation are being employed for preserving sperm fertilizing capacity (3–5). Therefore, methods for predicting and assessing sperm function are of great relevance to ensure optimal fertilization outcomes, which may have important economic and ecological implications. The articles published in this Research Topic address different perspectives related to sperm analysis and preservation across different animal species.

A preliminary assessment of male reproductive ability in domestic animals is usually carried out by the evaluation of some anatomical characteristics (e.g., testes size) and health status (6). For AI purposes, basic semen analyses should be conducted to filter out low quality seminal samples such as those with reduced sperm motility and concentration. The prediction of sperm quality before semen collection can lead to early detection of superior sires and help to establish their optimal semen collection regime. In rams, the use of testicular ultrasonography (Montes-Garrido et al.) is a non-invasive and predictive tool for estimating variations in the sperm quality of sires subjected to different frequencies of semen collection. Semen, besides spermatozoa, also consists of seminal plasma (SP) that not only acts as a transport medium for sperm cells within the female reproductive tract but also influences sperm function and offspring development (7, 8). Recently, the extracellular vesicles (EVs) present in the SP have gained special attention (9) as they have been linked to sperm function (10). Thus, the characterization and identification of the miRNA expression profiles in the SP-EVs isolated from fertile and sub-fertile males could serve as fertility biomarkers (11). Moreover, the importance of EVs does not lie solely in their characterization, but also in their application in ARTs. For this reason, it is important to know if the cryopreservation process as well as the addition of extenders, with animal or vegetal lipid content, could interfere in the correct profiling of these EVs for being used as biomarkers of sperm quality and fertility (Capra et al.).

Despite the advances reached during the last decades, sperm damage induced by semen storage still represents a common and almost unavoidable side effect of semen handling and preservation procedures. Genetics and ejaculate traits have been shown to be implied in the sperm ability to withstand the preservation protocols (12, 13), but there are still gaps to fill to identify the causes of a poor sperm tolerance to semen storage. For instance, it has been reported that the differences observed in sperm cryotolerance in goats may be related to certain amino acids and metabolic intermediates present in the SP, which can therefore be used as potential biomarkers of sperm freezability in this species (Xu et al.).

Yet, several procedures (e.g., centrifugation, dilution, addition of cryoprotectants) and factors (e.g., osmotic and thermal changes) that occur before, during, and after sperm storage can induce a state of oxidative stress that finally impairs the sperm function (14). For this reason, the characterization and localization of reactive oxygen species (ROS) by using different fluorescent probes are crucial to study their effects on sperm function (Palacin-Martinez et al.). A plethora of antioxidants have been developed to scavenge specific ROS such as those produced in the mitochondria. The mito-TEMPO, a mitochondria-targeted antioxidant, has been successfully tested in tomcat (Ali Hassan et al.) and bull (Elkhawagah et al.) spermatozoa during the cryopreservation process at a concentration ranging from 10 to 55 μM. While in the tomcat spermatozoa this antioxidant preserves the acrosome structure, in bull it also improves sperm kinetics, organelles and DNA integrity, cleavage and blastocyst formation rates. Thanks to their antioxidant and antimicrobial properties, natural compounds also represent a promising supplement for semen extenders that can be economically and environmentally sustainable (15, 16). Thus, several natural-based products [i.e., alpha-lipoic acid (Sun et al.), myo-inositol (Jawad et al.), phosphorus/vitamin B12 (Suwimonteerabutr et al.), and selenium (Paul et al.)] have been used as alternative additives in the preservation of rooster (Suwimonteerabutr et al.), ram (Sun et al.), and boar (Jawad et al.; Paul et al.) semen with positive effects on sperm function (e.g., decreased ROS and lipid peroxidation levels during semen preservation) and higher pregnancy rates. Once the sperm is deposited into the female genital tract, either by natural mating or AI, neutrophil extracellular traps (NETs), triggered by spermatozoa, are recruited into the female reproductive tract often causing reduced sperm function and fertilizing ability (17, 18). The synthetic glucocorticoid methylprednisolone (MPS) has shown promising results inhibiting the adverse effects of lipopolysaccharide-induced polymorphonuclear neutrophils on boar spermatozoa (Li et al.). Additionally, MPS supplementation also exerts positive effect on sperm function during liquid semen preservation at 17°C and acts as a ROS scavenger.

Even though the cryopreservation is considered the gold standard for long-term sperm storage, the use of liquid nitrogen that is required for this purpose shows a considerable carbon footprint (manufacture and transportation) and is also associated with large amount of waste. Alternatively, the freeze-drying is an interesting method for long-term sperm storage at room temperature and at 4°C. Despite freeze-dried spermatozoa are motionless and dead (conventional sense), their nuclei can support normal embryonic development after being injected into oocytes (19). In the present issue, a technique known as vacuum-drying encapsulation (VDE), which was originally developed for nucleic acid conservation in anhydrous state, has been successfully adapted to ram spermatozoa. Compared to canonical lyophilization, VDE better preserves the structural and fertilization potential of ram spermatozoa stored for 2 years both at room temperature and at 4°C (Palazzese et al.).

Alongside the advancements in ARTs highlighted in this Research Topic, improvements in sperm function analyses are essential for predicting fertility outcomes. Under this perspective, multi-parametric flow cytometry analyses have become an interesting tool for determining sperm quality parameters during the cryopreservation process, but they miss other important parameters such sperm morphology. The use of hybrid imaging flow cytometry, which combines flow cytometry and light microscopy features, may help to standardize sperm quality assessment protocols (Umirbaeva et al.).

Taking together, this Research Topic provides a series of articles dealing with novel techniques and analyses for predicting sperm quality and improving semen preservation protocols with the ultimate goal of maximizing ARTs' efficiency.
